# Intracellular cytokines in peritoneal leukocytes relate to lifespan in aging and long-lived female mice

**DOI:** 10.1007/s10522-024-10110-0

**Published:** 2024-05-15

**Authors:** Irene Martínez de Toda, Judith Félix, Estefanía Díaz-Del Cerro, Mónica De la Fuente

**Affiliations:** 1 Unit of Animal Physiology, Department of Genetics, Physiology, and Microbiology, Faculty of Biological Sciences, Complutense University of Madrid, José Antonio Novais, 12, 28040 Madrid, Spain; 2 Institute of Investigation Hospital 12 Octubre (imas12), 28041 Madrid, Spain

**Keywords:** Immune cells, Immunosenescence, Inflammation, Longevity, Flow cytometry

## Abstract

Peritoneal immune cell function is a reliable indicator of aging and longevity in mice and inflammaging is associated with a shorter lifespan. Nevertheless, it is unknown if the content of cytokines in these immune cells is linked to individual differences in lifespan. Therefore, this work aimed to investigate different peritoneal leukocyte populations and their content in intracellular pro-inflammatory (TNF and IL-6) and anti-inflammatory (IL-10) cytokines by flow cytometry in adult (10 months-old, n = 8) and old (18 months-old, n = 20) female Swiss/ICR mice. In addition, old mice were monitored longitudinally throughout their aging process, and the same markers were analyzed at the very old (24 months-old, n = 8) and long-lived (30 months-old, n = 4) ages. The longitudinal follow-up allowed us to relate the investigated parameters to individual lifespans. The results show that long-lived female mice exhibit an adult-like profile in most parameters investigated but also display specific immune adaptations, such as increased CD4+ and CD8+ T cells containing the pro-inflammatory TNF cytokine and CD4+ T cells and macrophages containing the anti-inflammatory cytokine IL-10. These adaptations may underlie their exceptional longevity. In addition, a negative correlation was obtained between the percentage of cytotoxic T cells, KLRG-1/CD4, large peritoneal macrophages, and the percentage of CD4+ T cells containing IL-6 and macrophages containing IL-10 in old age and lifespan, whereas a positive correlation was found between the CD4/CD8 ratio and the longevity of the animals at the same age. These results highlight the crucial role of peritoneal leukocytes in inflammaging and longevity.

**Supplementary Information:**

The online version contains supplementary material available at 10.1007/s10522-024-10110-0.

## Introduction

The immune system is an excellent marker of health, given its essential protective role from outer and inner potential threats to our health. Thus, when it functions properly, the individual's health is ensured. However, the function of this system deteriorates with age, a complex process known as immunosenescence that involves several changes at the cellular and humoral levels. This complex reorganization involves some cells and functions being augmented, others decreasing, and others remaining constant (De la Fuente and Miquel 2009). Some innate and adaptive immune functions have been shown to decline with age, while healthy, long-lived individuals display an adult-like function of those cells (Martinez de Toda et al. 2016), which has been thought to be the underlying reason for their ability to reach a very advanced age in healthy condition (Franceschi et al. 1995; Alonso-Fernandez et al. 2008). Long-lived individuals are able to escape the most common age-related diseases and they appear to be effectively protected from cancers. However, the mechanisms that underlie this protection are quite complex and still largely unclear (Salvioli et al. 2009). One possibility could be that remodeling of the immune system throughout the aging process could follow a different pathway in individuals who do achieve longevity than in those who do not.

Regarding immune cell subpopulation changes with aging, it is known that aging is accompanied by a reduction in the number of naïve CD4+ and CD8+ T cells together with an increment in increased memory CD4+ or CD8+ T cells (Qin et al. 2016). The age-related decrease in naïve T cells is thought to be a consequence of the age-associated thymic involution and replacement of functional tissue by fat (Starr et al. 2003), and it can underlie the deteriorated immune response in elderly individuals. Moreover, the age-related accumulation of late-stage memory cells seems to result from lifetime exposure to persistent infections, most commonly to cytomegalovirus. However, it is unknown if the accumulation of memory cells with age is a consequence of aging per se or if it is an advantageous adaptation in older adults to provide continuous protection against pathogens encountered early in life (Pawelec 2018). To shed light on the pro- or anti-longevity age-related changes in the elderly, it is necessary to correlate these changes to mortality. One of the most established immunosenescent changes is the inverted CD4/CD8 ratio, which has been postulated as a driver of comorbidities and mortality (Pawelec et al. 2001; DelaRosa et al. 2006). Most human studies have only examined peripheral blood leukocytes. Still, most immune cells are contained in other tissue compartments, which probably do not exhibit the same patterns of cell subset distribution as the blood (Thome and Farber 2015). It is unknown if the changes described in circulating blood leukocytes also occur in immune cells from the peritoneal cavity, in which the abovementioned link between immune function and longevity has been established in mice (Martinez de Toda et al. 2016). Another marker that has been reported to experience an age-related increase in T cells is the expression of the co-inhibitory receptor killer-cell lectin like receptor G1 (KLRG-1). Moreover, it has been suggested that KLRG-1 signaling may be responsible in part for the age-related defects observed in highly differentiated T cells (Henson and Akbar 2009). Nevertheless, it is unknown if the expression of this marker experiences age-related changes in peritoneal T cells and if those changes can be related to longevity.

Moreover, the peritoneal cavity contains macrophages, which are thought to play an essential role in orchestrating the immune response and can modulate the aging rate of the individual by their production of oxidant and inflammatory compounds (Vida et al. 2017; Martinez de Toda et al. 2021). Only a few studies have investigated age-related changes in subpopulations of peritoneal leukocytes. One study reported that old mice display a lower percentage of macrophages than long-lived mice, whereas no differences were found between adult and old mice (Arranz et al. 2010). On the contrary, another study reported that old mice show a higher percentage of macrophages but lower B and T lymphocytes than adult mice (Vida et al. 2017). Nevertheless, the link between these changes in peritoneal leukocytes and lifespan has not been previously investigated. In addition, regarding peritoneal macrophages, some studies have reported that they can be divided into two different subpopulations, which have been termed large peritoneal macrophages (LPM) and small peritoneal macrophages (SPM). They have been found to display different response patterns in terms of nitric oxide production in response to lipopolysaccharide (LPS) (Ghosn et al. 2010). However, how these populations change due to aging and how they relate to lifespan is unknown.

Moreover, not all aspects of immunity decrease with age as aging is accompanied by the establishment of a systemic low-grade chronic inflammation, which has been termed inflammaging (Franceschi et al. 2000; Fulop et al. 2023), characterized by elevated concentrations of mediators such as IL-6, TNF, and C Reactive protein (CRP). Inflammation strongly predicts morbidity and mortality, and chronic inflammation is detrimental to a functioning immune system. Several different sources of this age-related increased inflammation have been stated, including the age-related increase in senescence-associated secretory phenotype (SASP) cells, elevated damage-associated molecular patterns (DAMPs), increased gut permeability and the failure of the resolution of inflammation in old subjects, among others (De Maeyer and Chambers 2021). Moreover, immune cells, especially macrophages, have been proposed to facilitate the inflammaging phenomenon (De Maeyer and Chambers 2021; Fulop et al. 2023) and modulate the oxidative-inflammatory stress of the individual and, consequently, the aging rate (Martinez de Toda et al. 2021). In previous studies from our research group, we have found that peritoneal leukocytes from old mice show a higher release of pro-inflammatory cytokines, such as IL-1β, IL-6, and TNF, compared to when they were adults. Strikingly, we found that long-lived mice showed an even higher basal release of pro-inflammatory cytokines, such as IL-1β and IL-6 than when they were old (Martinez de Toda et al. 2017). In addition, they were found to release high levels of the anti-inflammatory cytokine IL-10 in unstimulated conditions, even higher than when they were adults, which could be the underlying mechanism for reaching healthy longevity (Martinez de Toda et al. 2017).

Thus, to ascertain which changes in immune cell subpopulations take place with aging in the peritoneal cavity, the major peritoneal leukocyte subpopulations: T lymphocytes, B lymphocytes, macrophages, T-helper (CD4+), T cytotoxic (CD8+), KLRG-1/CD4, KLRG-1/CD8, small peritoneal macrophages (SPM), and large peritoneal macrophages: LPM) will be measured in adult, old, very old, and long-lived mice. Moreover, the percentage of cells containing pro-inflammatory (IL-6 and TNF) and anti-inflammatory (IL-10) cytokines will be measured in CD4+ and CD8+ T cells, as well as in macrophages in adult, old, very old, and long-lived mice. Moreover, to further shed light on the specific contribution of the investigated markers in aging and longevity, the values at old age will be related to individual achieved lifespans.

## Materials and methods

### Animals

We used 28 female outbred Swiss/ICR mice (*Mus musculus*) purchased from Janvier Labs (France) of different ages, namely adult (10 ± 1 month, n = 8) and old (18 ± 1 month, n = 20). The group of old mice was monitored during their aging process, and data acquisition was performed in the survivors at the very old (24 ± 1 month, n = 8) and exceptionally long-lived (30 ± 1 month, n = 4) ages. The exceptionally long-lived mice had naturally achieved healthy and successful aging since the average lifespan for Swiss/ICR females in our animal house is 91.9 ± 5.6 weeks (Guayerbas et al. 2002; Arranz et al. 2010). Mice were housed at 4–5 per cage and maintained in standard laboratory animal conditions for pathogens, temperature (22 ± 2 °C), and humidity (50–60%) on a 12/12 h reversed light/dark cycle (lights on at 20:00 h) to avoid circadian interferences. Mice had access to tap water and standard pellets (Panlab, Spain) ad libitum. Individual lifespans of the old group of mice were monitored. The protocol was approved by the Experimental Animal Committee of Complutense University of Madrid (Spain) (PROEX 224.0/21). Animals were treated according to the European Community Council Directives ECC/566/2015 and ARRIVE guidelines.

### Collection of peritoneal leukocytes

Peritoneal suspensions were collected from 08.00 to 10.00 h, during the highest peak of activity of the animals, to minimize circadian variations on immune system measurements due to daily changes in corticosterone levels. Mice were not sacrificed. They were held by the cervical skin, the abdomen was cleansed with 70% ethanol, and 1 ml of sterile Hank’s solution, previously tempered at 37 °C, were injected intraperitoneally. After massaging the abdomen, 80% of the injected volume was recovered. The cellular suspensions were washed at 400×*g* for 10 min and adjusted to 2 × 10^6^ leukocytes/ml in PBS with BSA 1% (Sigma, St Louis, USA). Cellular viability was checked by the Trypan Blue (Sigma) exclusion test, and only suspensions with cell viability higher than 99 ± 1% were used for experiments.

### Flow cytometric analysis of peritoneal leukocyte populations and cytokine content

Peritoneal leukocyte suspensions were adjusted to 2 × 10^6^ cells/ml. 96-well V-bottom plates were used for antibody labeling. 200 µl of leukocyte suspensions were added to each well, the plate was centrifuged, and the supernatant was removed. The pellet cell was resuspended in a Cell Staining Buffer (Biolegend, USA), and specific antibodies for extracellular labeling were added to each well and incubated for 30 min in the dark. Then, plates were washed twice to remove excess antibodies, and a Cell Fixation Buffer (Biolegend, USA) was added. In addition, for intracellular labeling, an Intracellular Staining Permeabilization Wash Buffer 1 × (Biolegend, USA) was added. Subsequently, cytokine intracellular antibodies were added and incubated for 30 min in the dark. Finally, cell fixation buffer was added, and all wells' content was transferred to cytometry tubes. The antibodies used for extracellular labeling were: PerCP/Cyanine5.5 anti-mouse CD45 (Ref. 103132; Clone 30-F11), PE anti-mouse F4/80 (Ref. 123110; Clone BM8), FITC anti-mouse/human CD11b (Ref. 101205; Clone M1/70), APC anti-mouse CD3 (Ref. 100236; Clone 17A2), FITC anti-mouse CD19 (Ref. 152404; Clone 1D3/CD19), PE anti-mouse CD4 (Ref. 100408; Clone GK1.5), PerCP/Cyanine5.5 anti-mouse CD8a (Ref. 100734; Clone 53–6.7), APC anti-mouse/human KLRG1 (MAFA) (Ref. 138412; Clone 2F1/KLRG1), PerCP/Cyanine5.5 anti-mouse I-A/I-E (MHC-II) (Ref. 107626; Clone M5/114.15.2). The following antibodies were used for intracellular staining: Alexa Fluor® 488 anti-mouse TNF-α (Ref. 506313; Clone MP6-XT22), APC anti-mouse IL-6 (Ref. 504508; Clone MP5-20F3), and Alexa Fluor® 647 anti-mouse IL-10 (Ref. 505014; Clone JES5-16E3). All antibodies were purchased from Biolegend, USA. Cells were acquired on the flow cytometer FACSCalibur instrument (BD Biosciences) with the support of the staff of the cytometry-associated research center of Complutense University of Madrid (Spain). The total number of cells collected was 2000. Cells were gated according to their forward- and side-scattering, and only the high expression of the corresponding leukocyte differentiation antigen or cytokine was taken as positive and analyzed by the FlowJoTM software and expressed as percentage (%) of CD3+ (T lymphocytes), CD19+ (B lymphocytes), F4/80+-CD11b+ (macrophages), CD4+/CD3 (T helper), CD8+/CD3 (T cytotoxic), KLRG-1CD4, KLRG-1CD8, F4/80^low^-MHC-II^high^ (small peritoneal macrophages: SPM), F4/80^high^/MHC-II^low^ (large peritoneal macrophages: LPM) cells with respect to the total number of cells present in the samples, and % of cells positive for TNF, IL-6 and IL-10 regarding the total number of cells from each leukocyte population.

### Statistical analysis

SPSS version 21.0 (Armonk, NY, USA) was used for the statistical analysis. Age-related differences were studied through non parametric test Kruskal–Wallis followed by Dunn’s test to identify which groups are different. Pearson´s correlation coefficients were calculated to analyze the relationship between the investigated parameters and lifespan. Figures were designed using GraphPad Prism 6.0.

## Results

**Fig. 1:**
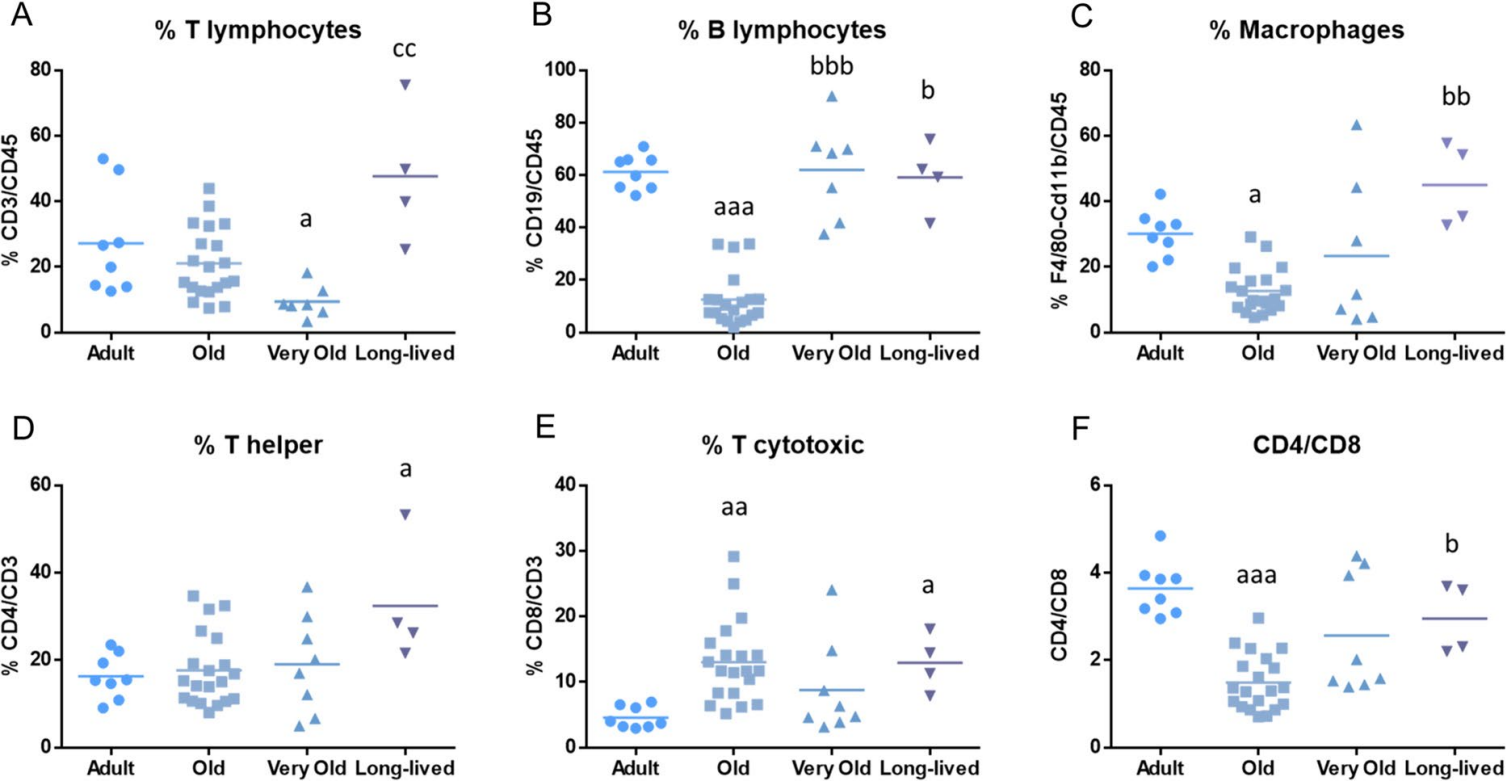
Age-related changes in different peritoneal leukocyte subpopulations. **A** T lymphocytes; **B** B lymphocytes; **C** Macrophages; **D** T helper; **E** T cytotoxic; **F** CD4/CD8 ratio. Graphs display the mean value of each age group. a: p < 0.05; aa: p < 0.01; aaa: p < 0.001 with respect to adult mice. b: p < 0.05; bb: p < 0.01 with respect to old mice. cc: p < 0.01 with respect to very old mice

First, the age-related changes in different immune cell subpopulations in the peritoneum of mice are shown in Fig. 1. Old mice were followed during their aging trajectories in a longitudinal study, whereas a group of adult mice was also included in the analysis to establish reference values. As can be seen in Fig. 1, old mice display a lower percentage of B lymphocytes (p < 0.001), macrophages (p < 0.05), a higher percentage of T cytotoxic lymphocytes (p < 0.01) and a lower CD4/CD8 ratio (p < 0.001) compared to adult mice. Very old mice show a lower percentage of T lymphocytes (p < 0.05) than adult mice but an increased percentage of B lymphocytes (p < 0.001) than that when they were old. Exceptionally long-lived mice display a higher percentage of T helper and T cytotoxic than adults (p < 0.05), and also a higher percentage of B lymphocytes (p < 0.05), macrophages (p < 0.01) and CD4/CD8 ratio (p < 0.05) than when they were old, and a higher percentage of T-lymphocytes (p < 0.01) compared to when they were very old.

**Fig. 2:**
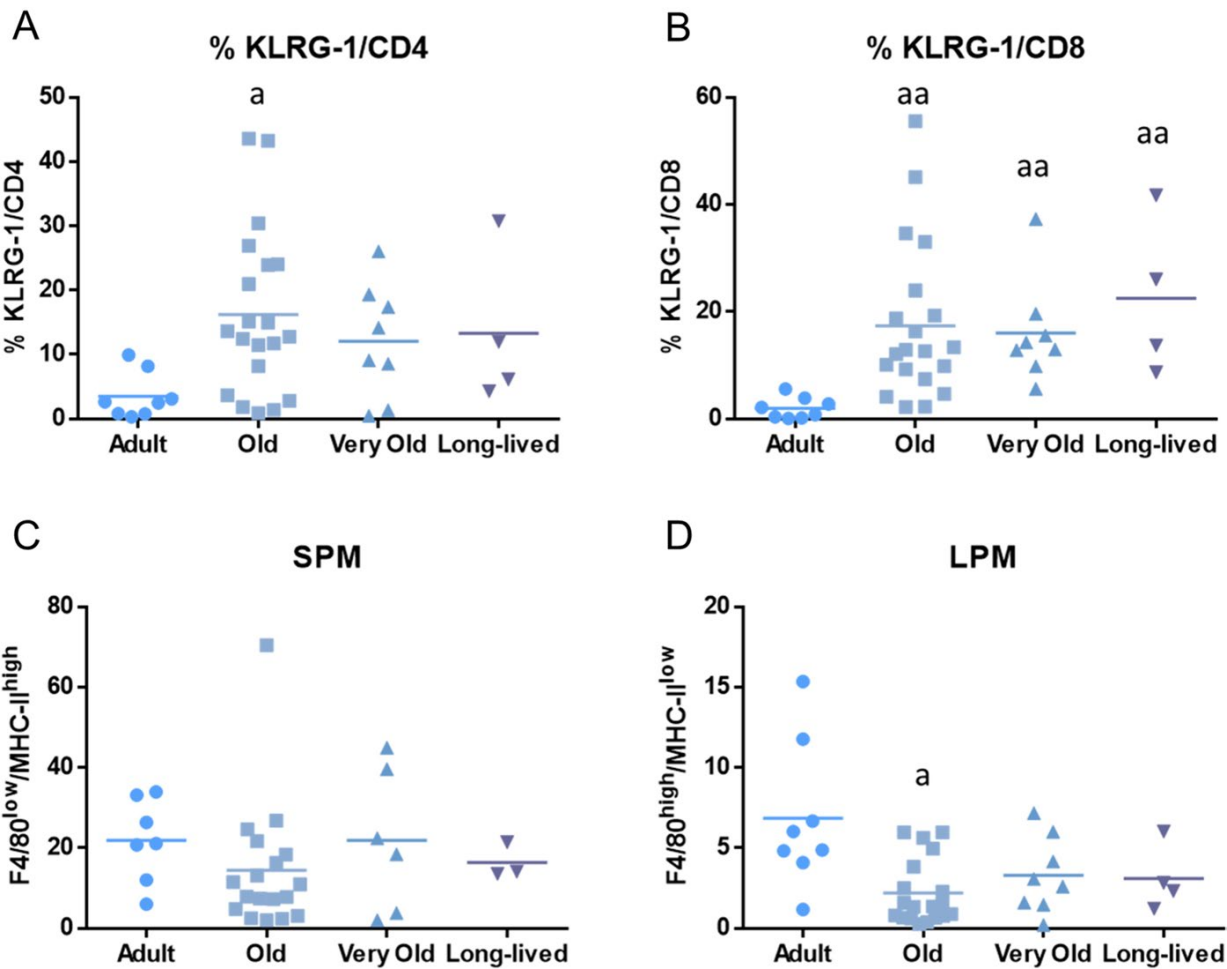
Age-related changes the expression of KLRG-1 in T cells and in the abundance of specific macrophage subpopulations. **A** KLRG-1/CD4; **B** KLRG-1/CD8; **C** small peritoneal macrophages (SPM); **D** large peritoneal macrophages (LPM). Graphs show the mean value of each age group. a: p < 0.05; aa: p < 0.01 with respect to adult mice. KLRG-1: co-inhibitory receptor killer-cell lectin like receptor G1

Moreover, age-related differences in the expression of KLRG-1, a marker whose expression has been related to the age-related impairment of T cells and in the percentage of two specific macrophage subpopulations identified as small peritoneal macrophages (SPM) and large peritoneal macrophages (LPM) are shown in Fig. 2. Old mice display a higher percentage of KLRG-1/CD4 (p < 0.05) and KLRG-1/CD8 (p < 0.01) T cells and a lower percentage of large peritoneal macrophages than adult mice (p < 0.05). In addition, very old mice and long-lived mice also display a higher percentage of KLRG-1/CD8 T cells (p < 0.01) than adult mice.

**Fig. 3:**
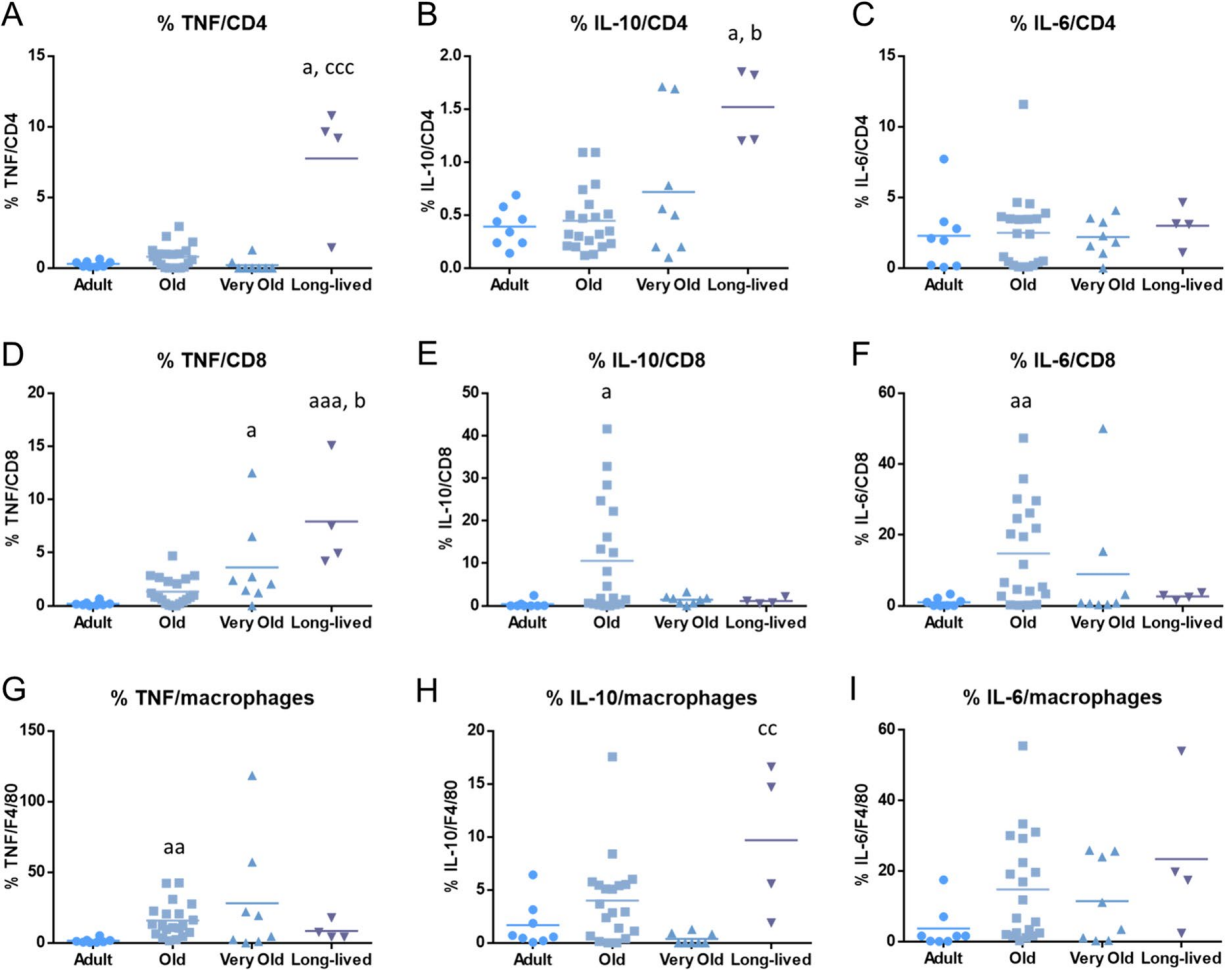
Age-related changes in the percentage of different leukocyte subpopulations with intracellular cytokines. **A** TNF/CD4; **B** IL-10/CD4; **C** IL-6/CD4; **D** TNF/CD8; **E** IL-10/CD8; **F** IL-6/CD8; **G** TNF/macrophages; H) IL-10/macrophages; I) IL-6/macrophages. Graphs show the mean value of each age group. a: p < 0.05; aa: p < 0.01; aaa: p < 0.001 with respect to adult mice. b: p < 0.05 with respect to old mice. cc: p < 0.01; ccc: p < 0.001 with respect to very old mice

In addition, the age-related changes in the percentage of different subsets positive for intracellular TNF, IL-10, and IL-6 expression are displayed in Fig. 3. Old mice show a higher percentage of CD8+ T cells containing IL-10 (p < 0.05) and IL-6 (p < 0.01) and a higher percentage of macrophages expressing TNF (p < 0.01) than adult mice. Moreover, very old mice show a higher percentage of CD8+ T cells containing TNF than adult mice (p < 0.05) and long-lived mice also display a higher percentage of CD4 containing TNF and IL-10 (p < 0.05) and a higher percentage of CD8+ T cells containing TNF (p < 0.001) than adult mice. Interestingly, long-lived mice also show an increased percentage of CD4+ T cells positive for IL-10 and CD8+ T cells positive for TNF than that when they were old (p < 0.05) and an increased percentage of CD4+ T cells positive for TNF (p < 0.001) and of macrophages expressing IL-10 (p < 0.01) than that when they were very old.

Moreover, to further investigate if the observed age-related changes are harmful, adaptive or neutral, old mice were individually monitored until their natural deaths and their individual lifespans were registered. Then, Pearson´s correlation coefficients were calculated between the values each mouse showed at the old age for each variable and their final achieved lifespan.

**Fig. 4:**
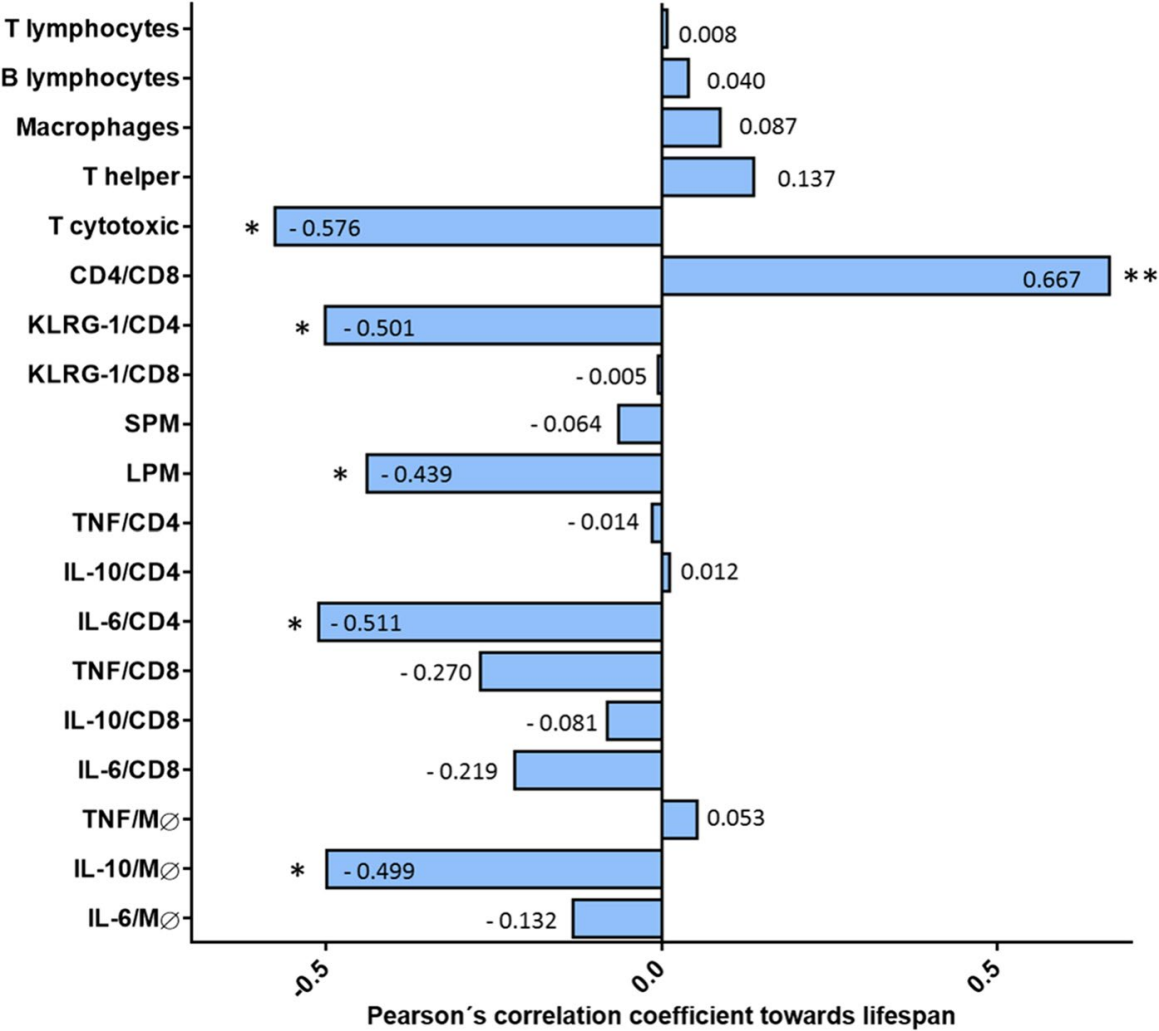
Pearson´s correlation coefficients between a series of immune cell markers at the old age and individual lifespans. *: p < 0.05; **: p < 0.01. *SPM* small peritoneal macrophages. *LPM* large peritoneal macrophages. *Mφ* macrophages

**Fig. 5:**
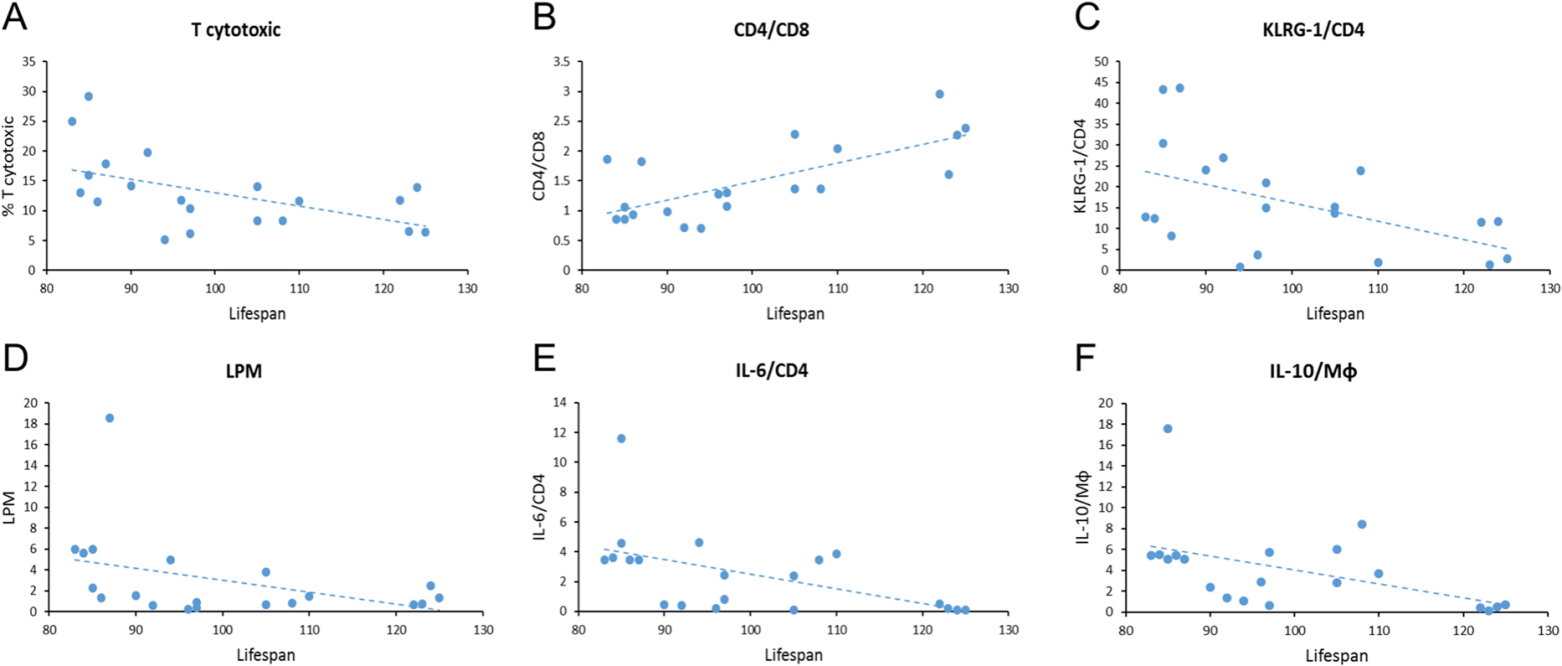
Graphical representation of statistically significant correlations between **A** T cytotoxic; **B** CD4/CD8; **C** KLRG-1/CD4; **D** LPM; **E** IL-6/CD4; **F** IL-10/Mφ and lifespan. *LPM* large peritoneal macrophages. *Mφ* macrophages

As can be seen in Figs. 4 and 5, there is a negative correlation between the percentage of T cytotoxic cells, KLRG-1/CD4 T cells, large peritoneal macrophages, the number of CD4+ T cells positive for IL-6 and the number of macrophages positive for IL-10 at the old age and lifespan (p < 0.05 in all cases) and a positive correlation between the CD4/CD8 ratio at the old age and final achieved lifespan (p < 0.01).

## Discussion

This is the first study that analyses the age-related changes of the principal leukocyte populations and their intracellular cytokine content from both innate and adaptive immunity branches from the peritoneum of mice, including animals that achieve exceptional longevity.

According to the present results, old mice show fewer B lymphocytes and macrophages than adult mice. Regarding age-related peritoneal macrophage changes, there is not a uniform consensus. Thus, it has been observed in mice that the percentage of these cells does not vary in old animals compared to adults (Herrero et al. 2002), while other studies have demonstrated an age-related decrease of peritoneal macrophages (Puerto et al. 2005; Arranz et al. 2010). Concerning B peritoneal lymphocytes, our results agree with a reduction in this population described in previous studies (Arranz et al. 2010; Vida et al. 2017). Nevertheless, our results should be interpreted with caution given that the adherent capacity of both macrophages and B lymphocytes has been reported to be increased in old mice (De la Fuente and Miquel 2009), which could have interfered with the cell number recovered from these populations. In addition, our results show that old mice show a higher percentage of cytotoxic T cells and a lower CD4/CD8 ratio than adult mice. These results agree with the typical immunosenescence changes described in peripheral blood leukocytes in mice and humans (Pawelec 2002; Bauer and De la Fuente 2016; Qin et al. 2016; Xie et al. 2017; Mittelbrunn and Kroemer 2021). We also found that old mice display an increased percentage of CD4+ and CD8+ T cells expressing KLRG-1 in the peritoneum, as has been reported in circulating leukocytes in mice and humans (Henson and Akbar 2009; Xie et al. 2017). Concerning the two different subpopulations of macrophages, old mice show a lower percentage of large peritoneal macrophages (LPM) compared to adult mice. It has been stated that both SPM and LPM populations coexist in the peritoneum. When there is an infection or an inflammatory insult, LPM numbers decrease drastically, given that they migrate to the *omentum* (Cassado Ados et al. 2015). LPM disappearance in response to inflammatory stimuli is accompanied by an increase in SPM and inflammatory monocytes, and it has been correlated with the renewal and improvement of immune conditions in the peritoneal cavity (Dos Anjos Cassado 2017). Thus, the lower percentage of LPM found in old mice could be the result of the age-related increase in inflammatory mediators.

In the study of aging, longitudinal approaches are of essential importance to ensure that reported trends are due to aging and not intergroup variability. Moreover, the longitudinal analysis of the age-related changes that exceptional long-lived individuals undergo during aging can help unravel essential mechanisms underlying the attainment of healthy longevity. In general, our results show that those animals that survive over the mortality peak of the general population (very old and long-lived) show closer values to the adult group than to those when they were old, in agreement with what we have previously observed in several immune function parameters (Martinez de Toda et al. 2016). At very old age, there was already an increase in the percentage of B lymphocytes and CD4/CD8 ratio. Moreover, at the long-lived age, there was a boost in the percentage of T and B lymphocytes, macrophages, T helper, and the CD4/CD8 ratio. Analyses of the immune cell populations in centenarians have concluded that these individuals display more naïve, activated/memory, and effector/memory CD4+ and CD8+ T cells (Bucci et al. 2014).

Among all the observed changes, the increased percentage of T helper cells seems to be a singular adaptation to this exceptional age, given that values are even higher than those found in the adult group. CD4 T cells represent a unique population crucial in achieving a regulated, effective immune response to pathogens and whose proper functioning is vital for survival (Luckheeram et al. 2012). Moreover, CD4 T cells have been reported to be able to function as cytotoxic cells in supercentenarians, and they are thought to underlie their ability to reach those exceptional ages by delaying the onset of age-related diseases and compression of morbidity (Hashimoto et al. 2019). Nevertheless, we cannot rule out that this increased percentage of T helper cells in long-lived mice could be the result of a mild form of leukemia or other tumors that cannot be easily identified. Still, we have previously shown that long-lived mice of this strain show preserved immune function parameters (Martinez de Toda et al. 2016) as well as inflammatory (Martinez de Toda et al. 2017) and oxidative stress markers (Martinez de Toda et al. 2020), which supports the idea of these changes being the result of a unique adjustment to this extreme age.

Although acute inflammation is vital for infection clearance or wound healing, chronic inflammation is detrimental to a functioning immune response and the individual’s health. Indeed, older people who have elevated circulating IL-6, CRP, TNF, IL-1β, or inflammasome-related genes have a higher chance of all-cause mortality (Harris et al. 1999; Bruunsgaard et al. 2003). Conversely, lower levels of inflammatory cytokines in the peripheral blood correlate with good health outcomes, longevity, and reduced risk of death in older adults (Arai et al. 2015). In our results, long-lived mice show an increased percentage of CD4+ and CD8+ T cells positive for TNF compared to when they were old and very old. Supporting our observation, it has been reported that paradoxically, long-lived individuals also display a systemic pro-inflammatory state that does not match their well-preserved immune system´s response (Martinez de Toda et al. 2016). Nevertheless, they also count on efficient anti-inflammatory networks termed anti-inflammaging that compensate for inflammaging (Franceschi et al. 2007) and enable an appropriate immune response (Serrano-Lopez et al. 2021). According to this hypothesis, we can see in our results that long-lived mice display an increased percentage of CD4 and macrophages positive for IL-10, which could be an adaptive compensatory mechanism to buffer the increased age-related inflammation. We have to keep in mind that we measured cytokine expression at basal conditions, that is, in the absence of stimulation, given that we wanted to investigate the contribution of peripheral immune cells to the establishment of inflamm-aging. Nevertheless, in a previous study from our research group, we analyzed the release of the anti-inflammatory cytokine IL-10 from peritoneal leukocytes both in basal condition and in response to concanavalin A and we found that peritoneal leukocytes from long-lived mice displayed a higher release of IL-10 cytokine, which could underlie their fine-tuned immune response (Martinez de Toda et al. 2017). In these experiments, cytokine concentrations were measured in culture supernatants of peritoneal leukocytes. Therefore, it was not possible to ascertain which immune cells are the ones that contribute the most to this release of cytokines. Thus, it should be investigated if CD4+ T cells and macrophages from long-lived mice also display a higher expression of these cytokines in response of a stimulus.

In a recent paper, it has been demonstrated that with aging there is a gradual functional decline that relates to mortality and other aging markers such as accumulation of senescent cells and the epigenetic clock (Marcozzi et al. 2023). We have previously analyzed the frailty phenotype in female mice of this strain and found that the individual frailty score of a given mouse was a relevant predictor of its lifespan (Martínez de Toda et al. 2018). Nevertheless, we have not investigated the relationship between the immune cell markers analyzed in the present study and functional capacity or frailty status of mice. Still, we believe that both processes are closely related and one can influence each other. In fact, both immunosenescence and inflammaging have been shown to be associated with the age-related development of frailty (Tran Van Hoi et al. 2023).

Finally, to shed light on the immunosenescent changes that may underlie reaching high longevity, the immune markers measured at old age in mice were related to their individual final achieved lifespans. This is the major strength of our study, and the use of peritoneal immune cells, which can be extracted from mice without the need to remove high volumes of blood, which may impact their homeostasis, made this approach possible.

We found a negative correlation between the percentage of T cytotoxic cells and senescent CD4 and a positive one between the CD4/CD8 ratio in old age and longevity. Our results agree with other studies performed in peripheral blood leukocytes, in which the CD4/CD8 ratio has been postulated as a predictor of mortality in humans (DelaRosa et al. 2006). Likewise, the negative correlation obtained between the number of senescent CD4 T lymphocytes and longevity agrees with a study reporting that an accumulation of senescent T lymphocytes accelerates the process of immunosenescence in mice (Badowski et al. 2014).

Strikingly, our study is the first to demonstrate a negative correlation between the percentage of large peritoneal macrophages (LPM) in old age and longevity. It has been stated that under homeostatic or resting situations, LPM account for 90% of the macrophages in the cavity, whereas small peritoneal macrophages (SPM) represent the remaining 10% (Cassado Ados et al. 2015). However, once activated in response to LPS or *Trypanosoma cruzi*, the subpopulation of SMP, which presents higher phagocytic activity and a higher release of IL-12, increases, which is believed to help promote the immune response (Ghosn et al. 2010; Dos Anjos Cassado 2017). Thus, the negative correlation between LPM and lifespan could indicate a diminished adaptation capacity to the well-known age-related increased inflammation in the peritoneal cavity.

In addition, we found a negative correlation between the percentage of CD4 T cells positive for IL-6 and the percentage of macrophages containing IL-10 in old age and longevity. The former would agree with the general hypothesis that the higher the inflammatory state in old age, the worse the functioning of the immune system and the lower the longevity. Accordingly, the circulating levels of IL-6 have been found to predict lifespan in older adults accurately (Puzianowska-Kuźnicka et al. 2016). Nevertheless, the fact that the higher the percentage of macrophages positive for IL-10 in old age, the shorter the lifespan seems paradoxical. We have previously identified that the higher the release of anti-inflammatory IL-10 from peritoneal leukocytes in old age, the greater the chance of achieving higher longevity (Martinez de Toda et al. 2019). A different study reported an increased release of IL-10 in exceptionally long-lived mice (Arranz et al. 2010). In another study, a lower release of IL-10 was found in the peritoneal leukocytes of old mice, while a higher one in those of long-lived mice compared to adults (Martinez de Toda et al. 2017). The previously observed lower levels in the extracellular media in old mice could be due to either a lower synthesis of this anti-inflammatory cytokine by immune cells or a lower release. The negative correlation obtained in the present work suggests that with aging, the release capacity of this cytokine is affected, accumulating intracellularly, since the greater the intracellular content of this cytokine, the shorter the longevity. Nevertheless, further research is needed to ascertain the mechanisms affected by age in immune cells' tightly regulated cytokine production response.

At last, it has been proposed that individuals with extended lifespans exhibit a decelerated aging process (Franceschi et al. 2018), thereby preserving optimal cellular function throughout the aging process. Nevertheless, our study’s findings contradict this notion, showing that long-lived individuals display immunosenescent trends when they are old. However, they can adapt and recalibrate these parameters at very old ages, showing optimal levels when they are long-lived. Our results support the hypothesis that individuals endowed with heightened biological plasticity or adaptive homeostasis (Pomatto and Davies 2017; Borras et al. 2020), manifesting in an increased proportion of CD4+ and CD8+ T cells expressing TNF or macrophages expressing IL-10, are more likely to achieve an extended lifespan. Nevertheless, due to the small sample size of mice that reached extreme longevity together with the batch effects and technical variability that affects intracellular staining of cytokines, the results of the present study should be validated using a larger number of long-lived mice.

Another limitation of our study is that these age-related changes and their link to lifespan were only investigated in female mice. Female mice were prioritized over males because they have a less aggressive behavior when housed together which reduces variability in immune cell activation states due to open wounds or scars, which could interfere with the obtained results. A possible alternative could be to house male mice individually, but it has been demonstrated that social isolation increases anxiety-like behaviors and affects the neuroimmunoendocrine communication (Cruces et al. 2014), which could also interfere with the immune markers measured in this study. Nevertheless, the findings reported in the present study should be validated in male mice to ascertain if it is a general adaptation of long-lived mice or it is sex-dependent.

In conclusion, our study proves that specific age-related changes in peritoneal leukocytes in old age are linked to individual differences in lifespan. This reinforces the hypothesis of the immune system's modulating role in an individual's aging rate (Martinez de Toda et al. 2021). Moreover, the present work highlights specific immune adaptations in long-lived individuals, which may underlie their achievement of a prolonged and healthy lifespan. At last, this research may contribute to fill in one of the seven knowledge gaps in modern biogerontology that have been recently described (Rattan 2024). In particular, this work provides one approach to help distinguishing between harmful, useful, and neutral changes occurring during ageing, that is by the study of the relationship between those changes at a given time point in the old age and lifespan. By these means, it is possible to identify those changes that can be adaptive or useful, as they are related to a longer and healthier lifespan, such as the specific immune cell changes reported here. The identification of these adaptive changes may contribute to developing strategies targeting these cell populations to strengthen immunity in elderly individuals and help them reach a healthy lifespan.

## Supplementary Information

Below is the link to the electronic supplementary material.

Supplementary file1 (TIF 4804 KB)
Supplementary Figure 1. Representative gating strategy. Peritoneal leukocytes were assessed within the FSC-height/SSC-height and they were selected in terms of size and complexity and previous experience of analyzing these cells. From this selected population, we further analyzed the expression of CD19+/CD45; Cd11b+/F4/80+ and CD3+/CD45, to quantify the percentage of B lymphocytes, macrophages and T lymphocytes, respectively. From the macrophages population selected, we further analyzed the expression of MHC-II and F4/80 to calculate the relative abundance of small peritoneal macrophages (SPMs) and large peritoneal macrophages (LPM) as well as the expression of intracellular TNF, IL-10 and IL-6. Similarly, form the T lymphocytes population selected, we further analyzed the expression of CD4+ and CD8+ T cells. Then, we selected CD8+ T cells and CD4+ T cells and in both populations we quantified the expression of KLRG-1 as well as the intracellular expression of TNF, IL-10 and IL-6.

## Data Availability

Data will be made available on request.
