# Supplementary figures and images for: Intracellular cytokines in peritoneal leukocytes relate to lifespan in aging and long-lived female mice
Source: Biogerontology. 2024 May 15;25(5):837–49. doi: 10.1007/s10522-024-10110-0 (PMC11374870; doi:10.1007/s10522-024-10110-0)

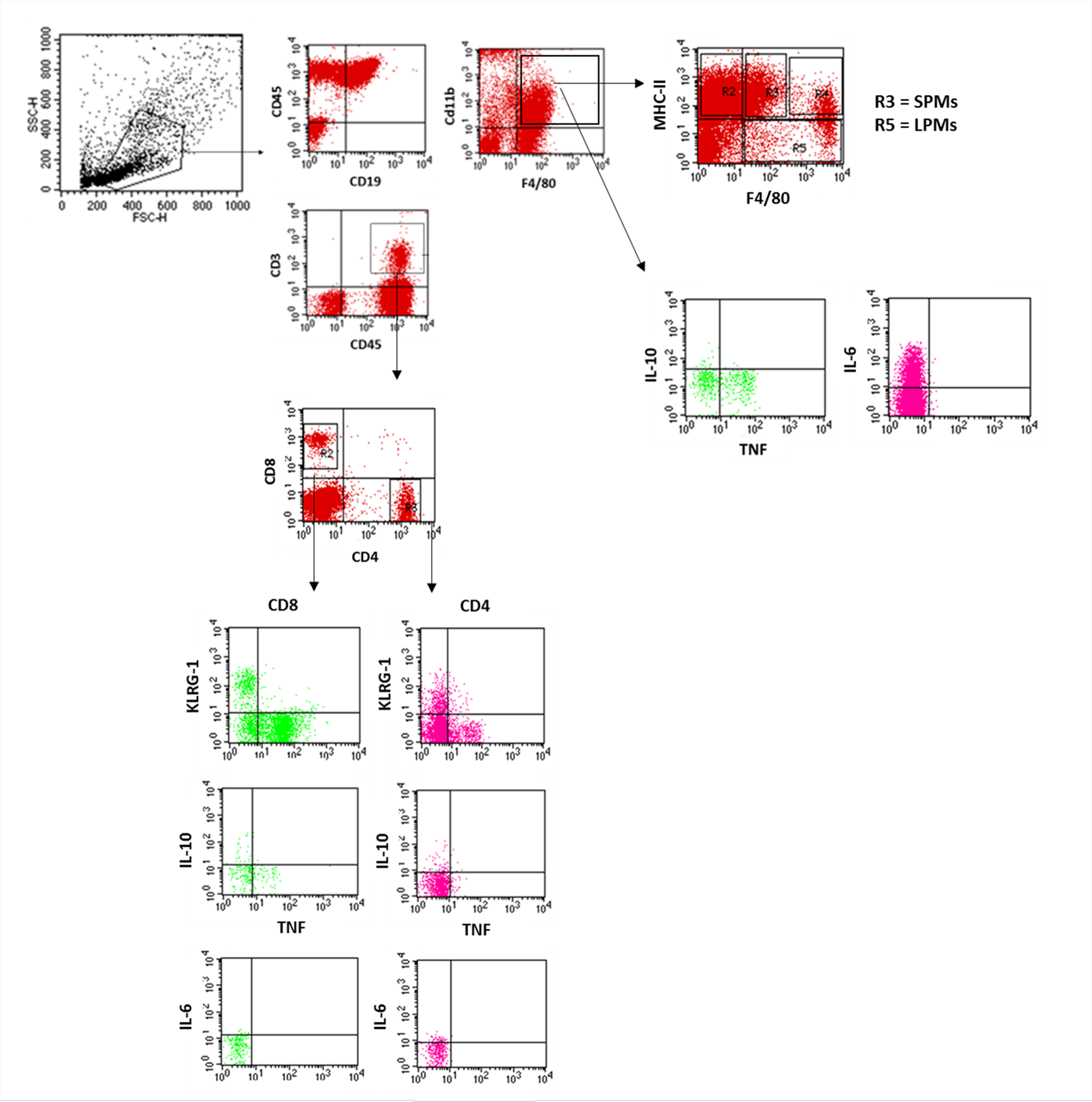

Supplement: Supplementary file 1 — Supplementary file1 (TIF 4804 KB) Supplementary Figure 1. Representative gating strategy. Peritoneal leukocytes were assessed within the FSC-height/SSC-height and they were selected in terms of size and complexity and previous experience of analyzing these cells. From this selected population, we further analyzed the expression of CD19+/CD45; Cd11b+/F4/80+ and CD3+/CD45, to quantify the percentage of B lymphocytes, macrophages and T lymphocytes, respectively. From the macrophages population selected, we further analyzed the expression of MHC-II and F4/80 to calculate the relative abundance of small peritoneal macrophages (SPMs) and large peritoneal macrophages (LPM) as well as the expression of intracellular TNF, IL-10 and IL-6. Similarly, form the T lymphocytes population selected, we further analyzed the expression of CD4+ and CD8+ T cells. Then, we selected CD8+ T cells and CD4+ T cells and in both populations we quantified the expression of KLRG-1 as well as the intracellular expression of TNF, IL-10 and IL-6. [file 10522_2024_10110_MOESM1_ESM.tif]
